# An aeroelastic wind energy harvester with continuous orbiting motion and no friction components

**DOI:** 10.1038/s41598-025-17512-1

**Published:** 2025-10-02

**Authors:** Petr Denissenko, Sam Tucker Harvey

**Affiliations:** School of Engineering, University of Warwick, Coventry, CV4 7AL UK

**Keywords:** Energy harvesting, Mechanical engineering, Fluid dynamics

## Abstract

A continuous-movement aeroelastic energy harvester with no friction parts is presented. Different from the commonly used vortex-induced vibration or galloping devices, the proposed energy harvesting system is constructed with a circular arc airfoil mounted to a flexible beam that follows a closed trajectory rather than oscillating linearly. The continuous motion of the airfoil results in the flow being fully attached, resulting in a greater efficiency than that of conventional oscillating wind energy harvesters. Experimental and numerical investigation has been conducted with the efficiency of energy conversion by the main element, the blade, measured to reach 3.5%.

## Introduction

Generation of energy from wind has been integral in the development of human civilisation and forms a key component in the response to climate change. Yet, at a small physical scale, wind energy may also prove to be an important energy solution. Autonomous electrical devices and their applications in sensor networks are set to revolutionize many industries, but current advances are limited by drawbacks of batteries and other energy storage devices. Small wind energy harvesters are well positioned to provide a solution to these problems.

The vast majority of modern wind energy devices are rotary in nature, converting kinetic energy into electrical with a rotating generator^1^. Although practical for large scale utility generation, the mechanical complexity in scaling these devices down and the presence of friction parts such as bearings makes them less suitable for harvesting energy for small electrical devices. These challenges are compounded in harsh and isolated environments. Oscillating aeroelastic energy harvesters, such as those operating due to vortex-induced vibration or the galloping instability of prismatic sections, are able to avoid many of these challenges with their mechanical simplicity and lack of friction parts. Such devices are generally composed of a bluff or streamlined body mounted to the end of a cantilever beam and are able to convert kinetic energy to electricity with the use of piezoelectric ceramics or via electromagnetic induction. A number of configurations have been suggested and optimised earlier^2–5^.

While the galloping instability of prismatic sections has long been studied as a phenomenon to suppress or avoid in many engineering applications^6–8^, its exploitation for wind energy harvesting has come more recently^9^. A key area of investigation has been the selection and optimisation of the tip geometry. Yang et al^10^ evaluated rectangular, triangular, and D-shaped cross-sections experimentally over a range of Strouhal numbers. Their analysis found the square prism to outperform the other geometries across the full range of velocities investigated. Together with simple base geometries, additions and modifications to these geometries have also been considered. The modification of the square prism with a wind-facing groove has been shown to result in a significant uplift of performance^11,12^, while the addition of small rods to the circular cylinder geometry has been shown to alter the response from one of Vortex Induced Vibration (VIV) to galloping, resulting in heightened harvesting performances^13^.

Yet, bluff body-based devices are limited by flow separation, where the wind energy is dissipated in a turbulent wake. This leads to low lift-to-drag ratios compared to the airfoil sections of rotating turbines, thus yielding far lower efficiencies. In a previous work^14^, the authors suggested an oscillating device including an airfoil-like geometry positioned perpendicular to the wind direction. The physical mechanism bears close similarity to that of the Well’s turbine, where the cross-wind motion of the aerodynamic profile creates angles of attack at which the flow is attached, hence yielding greater lift to drag ratios. A straightforward procedure of optimisation of the blade curvature^15^ led to the coefficient of performance of 8%. The device was however restricted by its oscillating trajectory, with the angle of attack changing sign each cycle, leading to the wind energy not being harvested over significant parts of the cycle. This issue also applies to flutter-based harvesters^16^, which produce zero energy when the angle of attack of the airfoil changes the sign.

Oscillating harvesters face further difficulty in the energy conversion from mechanical to electric power, with rectification frequencies as low as 1 Hz resulting in additional losses.

**Fig. 1:**
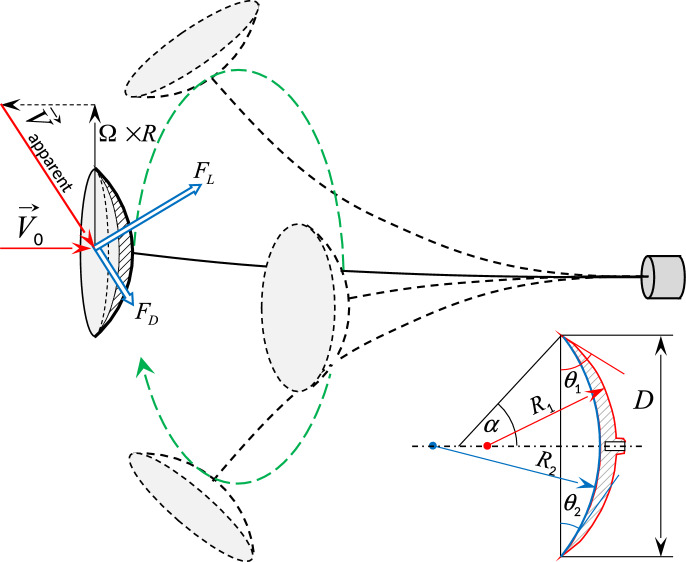
Orbiting blade energy harvester: axisymmetric airfoil mounted to an elastic beam. Lift and drag forces are shown acting on the circular blade. When the projection of the aerodynamic force on the blade direction of motion is positive, the blade orbiting accelerates. Observe that the apparent wind speed is formed by the free stream flow velocity and the blade’s own orbital motion. Inset: the circular ark airfoils are formed by two spheres of radii $R_1$ and $R_2$ and assigned nominal angles $\alpha$, which is associated with the angle of the leading edge to the disk plane. Angles of the windward and leeward surfaces to the blade plane are $\theta _2$ and $\theta _1$ respectively.

In this work, we remove the movement inhomogeneity by introducing an orbiting device, which follows a circular trajectory, rather than oscillating in a single cross-wind direction as shown in Fig. 1. This allows the tip geometry to maintain a constant angle of attack, sustaining flow attachment and hence a high lift to drag ratio. A prototype device is investigated through wind tunnel measurements of the efficiency of airflow-to-blade energy conversion, water flume visualisation of the attached flow, and Computational Fluid Dynamics (CFD), providing further insight into both the potential harvesting efficiency and the flow structure.

## Methods

### Experimental methods

For the experiments in air, a 1.2$\times$1.6 metre wind tunnel was used, where the orbiting blade was suspended by a mild steel beam of 1415 mm, and diameter of 5 mm. The beam was rigidly fixed to a 60 kg support to avoid energy loss through the movement of the root of the beam. Parameters of the 3d-printed circular blades of the diameter of 140 mm used are listed in the table 1. Blade trajectories were video recorded using a circular fluorescent marker.

**Table 1:** Blades geometry: nominal arch angles, used as names, masses, radii of spheres forming blade surfaces, and angles of the surfaces to the blade plane at the rim, as shown in the inset in Fig. 1.

Blade name	*m*	$R_1$, mm	$R_2$, mm	$\theta _1$	$\theta _2$
$35^\circ$	60	1057	1480	$42^\circ$	$30^\circ$
$45^\circ$	63	1120	900	$38^\circ$	$51^\circ$
$55^\circ$	68	920	805	$49^\circ$	$61^\circ$
$65^\circ$	74	804	745	$59^\circ$	$70^\circ$

For the experiments in water, a stainless steel beam of 900 mm length and 3 mm diameter was used with a blade of 53 mm diameter, formed by spheres of radii and angles at the rim to the blade plane of $R_1=42$ mm, $\theta _1=39^\circ$; $R_2=70$ mm, $\theta _2=22^\circ$.

Ink visualisation was performed by painting the blade with a lollipop-type mixture of sugar with methylene blue, liquefied by heating over a Bunsen burner. The applied mixture was considered thin and smooth enough to prevent disturbance of the boundary layer.

### Evaluation of harvesting performance

The efficiency of conversion of wind energy to mechanical energy was determined by evaluating the growth of total energy in the transient oscillation, as described in^15^. In air, where the kinetic energy of the fluid interacting with the airfoil is two-three orders of magnitude less than that of the airfoil itself, the blade orbits at the natural frequency of the blade-beam system and the total energy can be approximated as the sum of the beam and blade kinetic energy and the beam elastic energy:

1 $$\begin{aligned} E_{\textrm{T}} = \frac{m_t}{2} \left( {\dot{x}}^2 + {\dot{y}}^2 \right) + \frac{k_{\textrm{beam}}}{2} \left( x^2 + y^2 \right) , \end{aligned}$$

where

2 $$\begin{aligned} m_t = \left( m_{\textrm{beam, eff}} + m_{\textrm{blade}} \right) \end{aligned}$$

The effective mass of the beam $m_{\textrm{beam, eff}}$ corresponding to the kinetic energy of the bent beam, was calculated by solving the Euler-Bernoulli equation for the beam shape. In the wind tunnel experiments, $m_{\textrm{beam, eff}}=51$ g and is comparable with the weight of the blades themselves. Under the assumption of small deflections, the cantilever beam can be represented by the linear stiffness with the constant $k_{\textrm{beam}}$. Assuming the system nonlinearity is weak and significant timescale separation exists between beam natural vibrations and the blade orbiting frequency, the beam stiffness can be estimated from the blade trajectory from the frequency of disk orbiting *f*:

3 $$\begin{aligned} k_{\textrm{beam}} = 4 \pi ^2 m_{\textrm{t}} f^2 \end{aligned}$$

The energy harvesting performance can hence be estimated as the time derivative of the total energy. With the trajectory not being exactly circular, we use the cycle-averaged harvesting power ${\hat{P}}_{\text {h}}$, defined as derivative of the cycle-averaged total energy:

4 $$\begin{aligned} {\hat{P}}_{\textrm{h}} = \frac{d}{dt}{\hat{E}}_{\textrm{T}} \end{aligned}$$

To allow comparison with other wind energy devices, the non-dimensional coefficient of performance is adopted, which is the harvested power divided by the power delivered by the flow over the area swept by the blade, $A_s$:

5 $$\begin{aligned} C_{p0} = {\hat{P}}_{\text {h}}\frac{2}{\rho A_{s} U_{\infty }^3} \end{aligned}$$

Here $\rho$ is the air density.

The above considers the performance of the system, which harvests energy from the wind while damping it to the plastic and aerodynamic losses by the beam. To calculate the aerodynamic efficiency of the blade itself, we account for the damping in the cantilever beam supporting the blade. This was achieved by recording decaying oscillations of the blade in the absence of the wind to estimate the mechanical damping power $P_{\textrm{damping}}$ and then adding it to the measured power of the whole system, yielding efficiency of the harvesting element, the blade:

6 $$\begin{aligned} C_{p\,{\textrm{blade}}} = \left( {\hat{P}}_{\textrm{h}}+P_{\textrm{damping}}\right) \frac{2}{\rho A_{s} U_{\infty }^3}, \end{aligned}$$

### Numerical simulation

The flow around the orbiting blade that orbits around a circular trajectory was simulated using Star CCM+ commercial CFD solver. The exact shape of the supporting beam was found by numeric solution of the Euler-Bernoulli beam bending equations with boundary conditions corresponding to one end fixed and the other end holding the orbiting blade with the correction introduced by beam inertia. The solution for the angle of the blade end to the axis was used to correctly incline the blade to the free stream flow.

To avoid computationally intensive time-resolving modelling of an orbiting object, the following trick was used: a steady simulation was set up in a cylindrical domain, with a frame of reference rotating with the blade orbiting rate $\Omega$. In this frame of reference, the blade is stationary and rotates around its own axis with the angular speed $-\Omega$, which was used to set the no-slip boundary conditions.

**Fig. 2:**
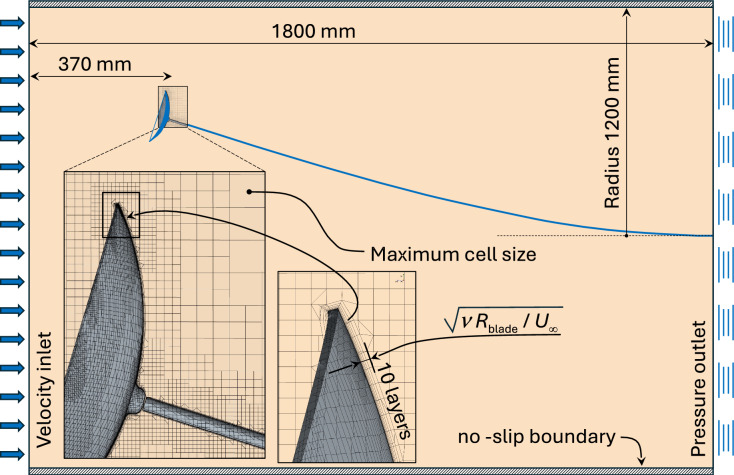
Cylindrical domain and the computational mesh for CFD simulations in air. An inset shows mesh around the blade. The maximum cell size, undistinguishable in the main figure, is indicated in the inset. The total thickness of the prism layers is comparable to the thickness of the viscous boundary layer at mid-blade.

Ideal gas and incompressible liquid Reynolds Averaged Navier Stokes (RANS) simulation were set up. At the flow speeds considered, not exceeding 5 m/s, compressibility and thermal effects are not significant. A trimmed cell mesh was used with 10 prism layers at the blade surface. The total thickness of the prism layer and the maximum cell size near the blade was not exceeding 1/150 of blade diameter. The maximum cell size far from the blade was not exceeding 1/10th of the blade diameter. Numerical studies of flows around disk-shaped objects with cavities (such as Frisbees) have been performed in more irregular geometries^17^, where flow separation occurs. In the regimes used for energy harvesting in the current study, the flow remains attached to the circular arc airfoils, and boundary layers remain laminar, relieving the simulation of the flow around the blade from modelling turbulence, and therefore making results more reliable. The mesh for the air simulation is shown in Fig.2

Turbulence was modeled using the standard *k*–$\epsilon$ two-equation model, which estimates the turbulent viscosity based on the turbulent kinetic energy (*k*) and its dissipation rate ($\epsilon$). Since the flow remained attached along the blade surface and the blade-diameter-based Reynolds number did not exceed 40,000 in either air or water, the boundary layer was expected to remain largely laminar. As a result, the simulation was relatively insensitive to the choice of turbulence model.

A mesh sensitivity study was conducted by comparing simulations using a mesh 1.5 times coarser. The resulting difference in predicted quantities was within 1%, indicating good mesh independence. To evaluate the influence of turbulence modeling, particularly due to potential unsteady wake effects behind the stalk supporting the blade, simulations were performed assuming both laminar flow and using the *k*–$\epsilon$ turbulence model. The resulting differences in blade and beam moment predictions were less than 4%, demonstrating the robustness of the simulation results with respect to turbulence modeling assumptions.

## Results

### Orbiting circular arc airfoil

**Fig. 3:**
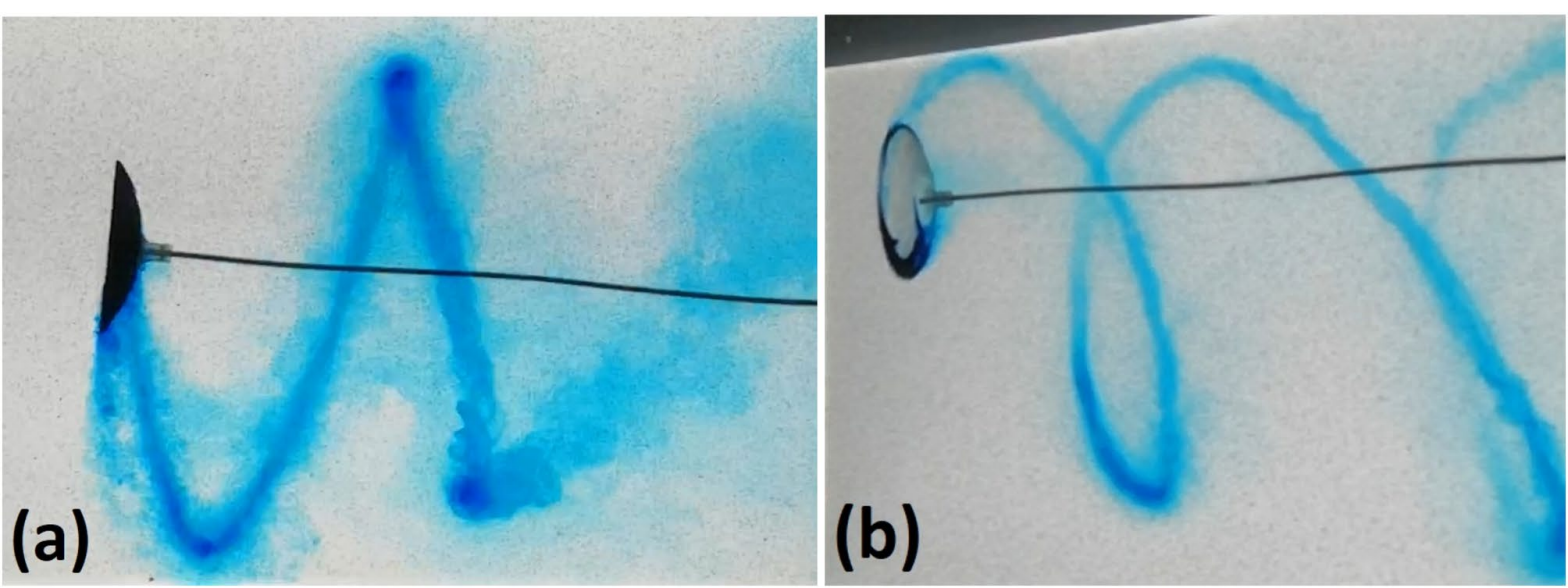
Ink visualisation illustrating flow attachment behind the orbiting blade (top) and the spiral trajectory of the orbiting blade and the wing-tip vortices (bottom). The blade is covered with a lolly pop-like crystallised sugar with Methylene blue added as a dye. The flow velocity is 0.35 m/s, blade diameter 53 mm, orbit diameter 130 mm $\left( \text {chord-based }{\textrm{Re}}\sim 4\cdot 10^4\right)$, orbiting frequency 1.65 Hz. The flow angle of attack on the plane of the blade rim is 41$\phantom{0}^\circ$ at the inner edge and 20$\phantom{0}^\circ$ at the outer edge. The blade is formed by the spheres of $\oslash$84 mm and $\oslash$140 mm, and the angles of the surfaces to the blade plane are 39$\phantom{0}^\circ$ and 22$\phantom{0}^\circ$.

The orbiting energy harvester is constructed by fixing a circular arc airfoil (the blade) perpendicularly to the end of a cantilever beam, which is mounted in an orientation parallel to the flow direction, as illustrated in Fig. 1. When the flow speed exceeds a critical value, the blade begins to orbit. The ink flow visualisation was performed to illustrate that the flow is attached to the blade (Fig. 3b) and that the motion is continuous (Fig. 3a). Videos of blade orbiting are available in the Supplementary Information, both at normal speed and slowed down, to illustrate that the flow remains attached to the surfaces.

The physics of acceleration of the blade, symmetric in the direction of motion, is similar to that of Wells turbine: due to inclination of the apparent wind speed, an accelerating component of lift force appears. The difference from the oscillating blade harvester described earlier^14^ is that now the motion is circular and with the constant speed as shown in Fig. 1. The crucial difference from Wells turbine is that the system contains no friction parts or bearings with orbiting motion occurring through bending of the supporting beam.

The system was evaluated experimentally in both air and water, with flow visualisation and limit cycle trajectories observed in water and the harvesting efficiency evaluated in air, where the blade inertia allows measurement of the energy growth rate^15^. These provided two points of comparison between the numerical and experimental results.

### Observations on evolution of the elliptical orbit

**Fig. 4:**
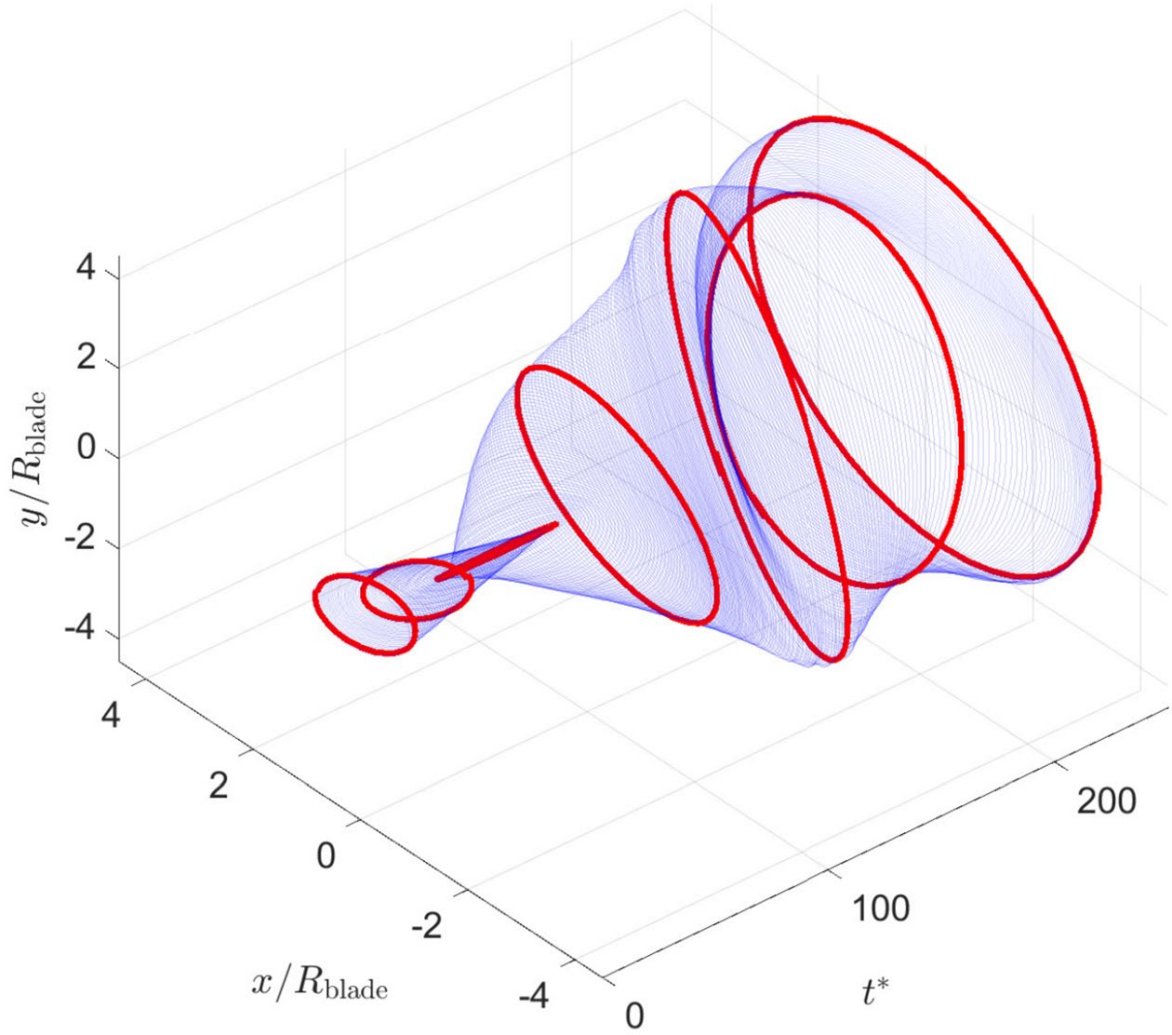
Trajectory of the disk centre (experiment r20). Seven orbiting cycles are highlighted in red to observe evolution of the shape of the disk trajectory ranging from nearly a circle to nearly a straight segment. The blade angle $\alpha =65^\circ$, wind speed 1.9 m/s.

**Fig. 5:**
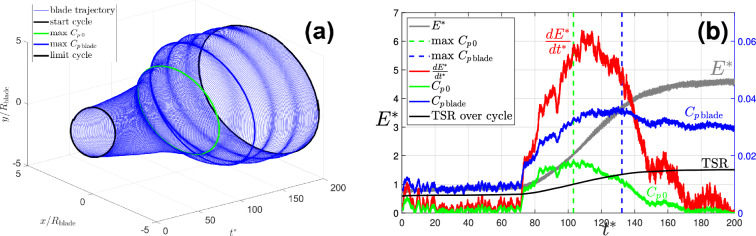
(**a**) Trajectory of the disk centre (experiment r13). The thick green line highlights the cycle with maximum $C_{p\,0}$ whose time location is marked by the green dashed line in (**b**). The thick blue line highlights the cycle where $C_{p\,{\textrm{blade}}}$ is evaluated, marked by the blue dashed line in (**b**) which corresponds to the tip speed ratio of 1.3. The blade angle is 35$\phantom{0}^\circ$, wind speed 1.7 m/s. (**b**) Growth of the total energy for (experiment r13) the 35$\phantom{0}^\circ$ blade, orbiting frequency 1.21 Hz. Maximum growth occurs at the elliptical trajectory with half-axes of 4 and 4.6 blade radii. The maximum $C_{p\,{\textrm{blade}}}$ is 3.5% is achieved at the tip speed ratio of 1.3. The energy is non-dimensionalized by $m_t U_\infty ^2/2$. Observe that the overall efficiency of the system $C_{p\,0}$ reaches maximum at a lower speed of the disk, when the damping in the supporting beam is lower. The cycles corresponding to maximum $C_{p\,0}$ and $C_{p\,{\textrm{blade}}}$ are highlighted by the corresponding colours in (**a**).

In our experiments, the trajectory of the blade was not always circular and exhibited complex dynamics, warranting further investigation. In different runs, oscillation amplitude grew differently in perpendicular directions, making the trajectory elliptic with the blade velocity vary substantially over an orbiting cycle, reaching maximum at co-vertices and minimum at vertices. An example trajectory illustrating the time varying properties of the ellipse in unstable runs is presented in Fig. 4. In extreme cases, the elliptical trajectory collapsed entirely and the blade oscillated rather than orbited. A system with similar behaviour has previously been observed by Lee and others^18^, who reported transition between planar and spiral trajectories of sinking circular disks. While in many cases the limiting cycle was far from elliptic, close to circular trajectories have been observed as shown in Fig. 5a.

### Harvesting performance

Harvesting performance has been measured in a set of experiments in air, presented in Table 2. We define two power conversion coefficients. The first, $C_{p\,0}$, corresponds to the amount of energy which can be extracted from the current system and is defined in accordance with equation (5). The second, $C_{p\,{\textrm{blade}}}$, is the derived performance of the blade itself, with no loss related to damping in the beam (6). The latter, higher performance, can be interpreted as the upper limit of performance of the mechanically optimised system.

**Table 2:** Efficiency of the wind energy conversion with $C_{p\,0}$ and $C_{p\,{\textrm{blade}}}$ defined by with equations (5) and (6). The Tip Speed Ratio, mean radius of the trajectory $r_{\textrm{traj}}$, non-dimensionalised by the blade radius $r_{\textrm{blade}}$, and the asymmetry (ratio of the axes of the elliptic trajectory) are shown at the point of maximum $C_{p\,\mathrm blade}$. Parameters of the experiment r13, highlighted in the table, have been used for the CFD simulation.

Run #	blade	$U_\infty$	$C_{p\,0}$	$C_{p\,{\textrm{blade}}}$	Tip speed ratio	$r_{\textrm{traj}}/r_{\textrm{blade}}$	Asymmetry of trajectory
	$\alpha$	m/s	Max	Max	at max $C_{p\,{\textrm{blade}}}$	at max $C_{p\,{\textrm{blade}}}$	
r14	35$\phantom{0}^\circ$	1.17	2.5 %	5.5 %	1.3	2.1	>10
r04	45$\phantom{0}^\circ$	1.66	3.0 %	4.5 %	0.79	2.6	12
r19	65$\phantom{0}^\circ$	1.65	2.6 %	3.9 %	0.57	1.9	>10
r02	45$\phantom{0}^\circ$	1.55	2.2 %	3.6 %	1.2	3.6	1.4
r13	${\textbf {35}}^{\circ }$	1.**70**	1.75 %	3.5 %	1.3	4.3	1.12
*CFD*	35$\phantom{0}^\circ$	1.70	*1.8* %	*3.3* %	1.3	4.3	*1*
r09	55$\phantom{0}^\circ$	1.56	2.2 %	3.5 %	0.68	2.2	4.6
r03	45$\phantom{0}^\circ$	1.63	2.3 %	3.5 %	1.2	3.9	1.32
r00	45$\phantom{0}^\circ$	1.77	2.0 %	3.2 %	0.91	3.0	1.35
r10$_{\textrm{flat}}$	55$\phantom{0}^\circ$	2.01	1.8 %	2.6 %	1.0	2.0	>10
r10	55$\phantom{0}^\circ$	2.01	1.4 %	2.3 %	1.0	3.3	1.7
r20	65$\phantom{0}^\circ$	1.89	1.1 %	2.1 %	0.64	2.5	4.4
r08	55$\phantom{0}^\circ$	1.77	2.7 %	0.4 %	0.68	1.3	5

Figure 5a shows an example trajectory of the disk centre, where the orbit grows from its initial size, converging to a limiting orbit. The cycle where the maximum aerodynamic performance $C_{p\,{\textrm{blade}}}$ is achieved is highlighted in red. The time series of the non-dimensional total energy in the system $E_{\text {T}}$, its derivative, and the coefficient of performance $C_{p\,0}$ and $C_{p\,{\textrm{blade}}}$ are plotted in Fig. 5. The markers show time instants where maximum energy growth rate and the maximum *Cp* are achieved. The latter maximum occurs before the maximum time derivative of total energy, because the swept area $A_s$ in (5), the area from which the wind energy is harvested, and hence the available wind energy, increases as the orbit grows.

To test the feasibility of our measurements, we have evaluated $C_{p\,{\textrm{blade}}}$ from data obtained by other groups on disks moving along a straight line. While not matching the exact disk shape, or accounting for the asymmetry between left and right sides (closer to and further from the axis of orbiting), and without matching the Reynolds number and therefore the magnitude of the skin friction, we have used values of lift and drag coefficients from studies related to sports equipment, such as Frisbee and throwing discus^19,20^. The obtained efficiencies of energy conversion $C_{p\,{\textrm{blade}}}$ do not exceeding single percents, depending on the studies chosen and the selected tip speed ratios. This is consistent with our experimental findings, while the dependence of the estimated values on the parameters of the disks invites a dedicated numerical investigation into streamlined concave geometries designed specifically for energy harvesting.

### Numerical simulation

In support of our interpretation of experimental results, a CFD simulation has been performed, resolving aerodynamic/hydrodynamic forces. The flow simulation in air was used to verify the value of measured $C_p$ for the case when the 35$\phantom{0}^\circ$ blade is orbiting at the radius of 30 cm with the frequency of 1.2 cycle per second: an experiment with the orbit closest to circular, r13 in Table 2, has been chosen. The moment of forces with respect to the system axis was calculated as 3.7 mN m for the blade and -1.7 mN m for the beam, leaving the total of 2 mN m, which generates mechanical power of 0.015 W corresponding to the efficiencies $C_{p0}=1.8\%$ and $C_{p\,{\textrm{blade}}}=3.3\%$. In water, geometric parameters of the experiment shown in Fig. 3 were used to set up the simulation, and the orbiting frequency $\Omega$ was varied to iteratively find the value when the total torque acting on the beam-blade system is zero, indicating the constant orbiting rate.

**Fig. 6:**
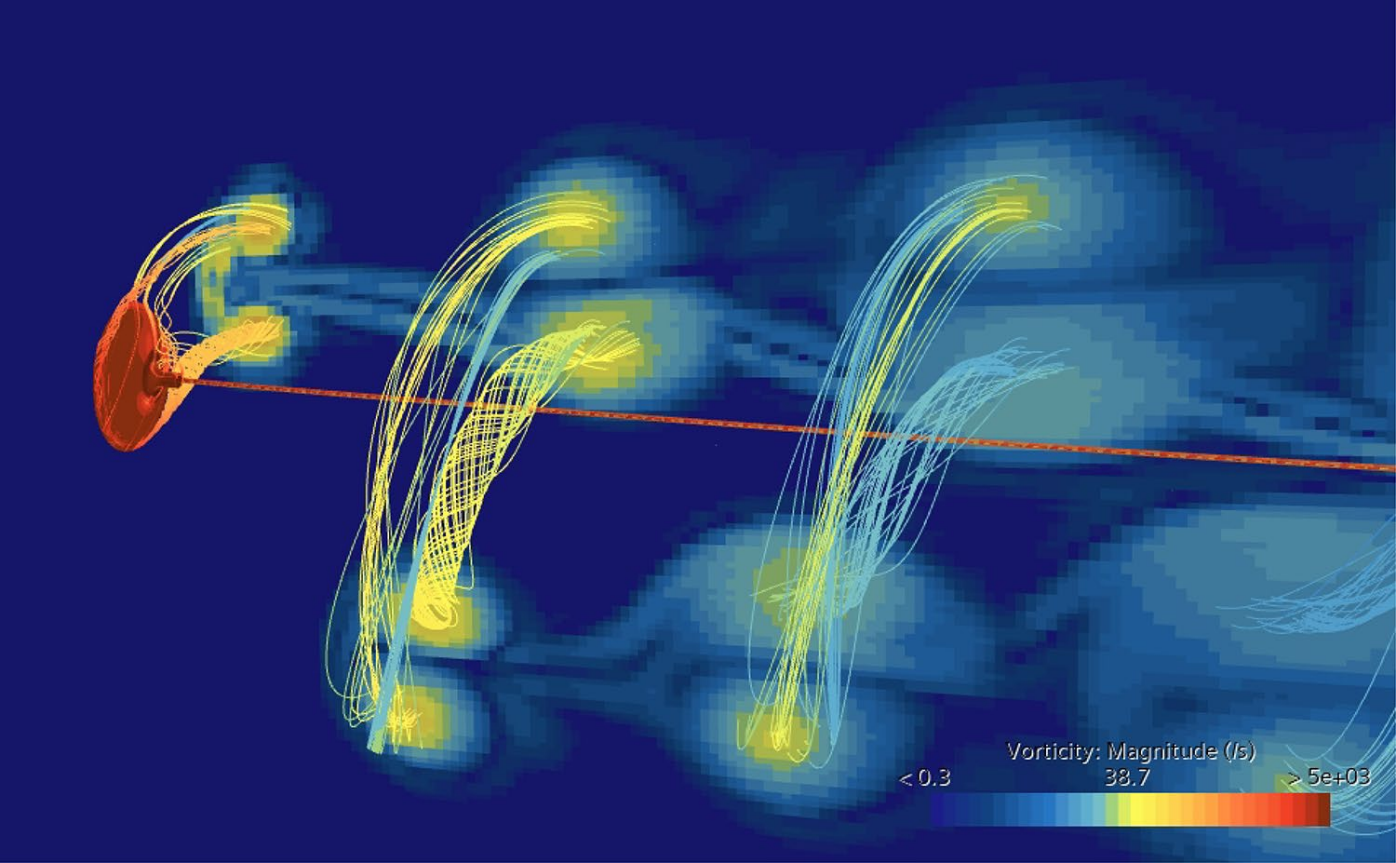
Streamlines and vorticity map produced by CFD simulation for the case shown in Fig. 3. Observe the pair of spiral vortices of the opposite sign shed by the blade edges and the wake created by the beam.

**Fig. 7:**
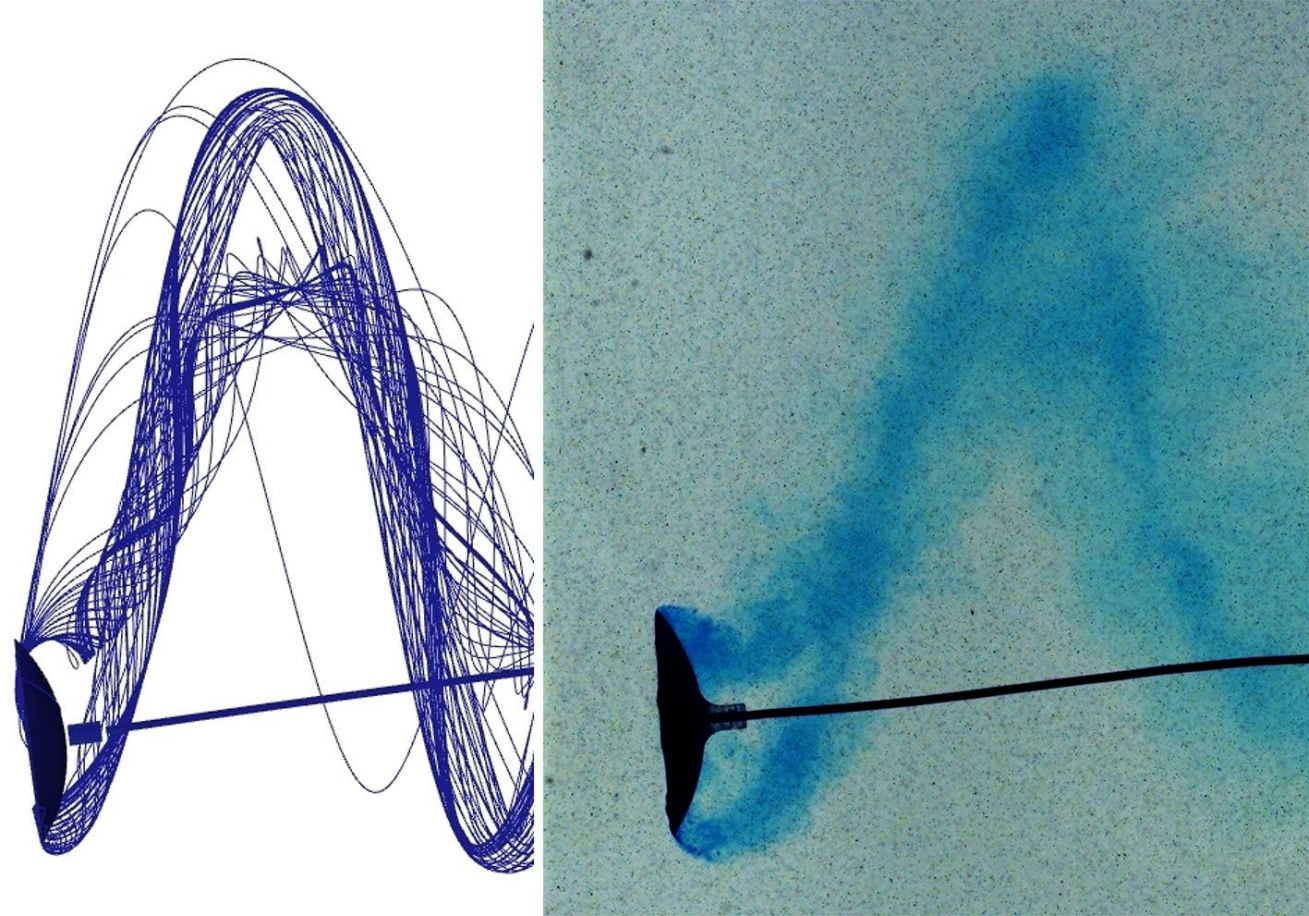
Streamlines produced by CFD and the ink visualisation for the case shown in Fig. 3, when the blade moves towards the observer. Note the vortices formed at the inner and outer edges of the blade.

While the CFD simulation did not account for plastic losses in the supporting beam, both the total system efficiency $C_{p0}$ and the blade’s own efficiency $C_{p\,{\textrm{blade}}}$ were in agreement with the measured value within 10%. In water, where the energy production is hard to measure experimentally because the density of water and the blade are comparable, and therefore transient regime is as short as several cycles, we have used the dimensions of the observed limiting orbit to calculate the orbiting frequency. The simulated streamlines are shown in Fig. 6 and compared with the ink visualisation in Fig. 7. The moment generated by the blade was exactly compensated by that of the beam drag at $\Omega =8.8$ while the experimentally observed value is $\Omega =10.4$. The discrepancy of 15% can be considered a reasonable agreement given the low-precision blade manufacturing, the non-uniformity and uncertain magnitude of the flow velocity in a teaching-grade water flume, the unknown mechanical losses in the beam, and the fact that simulated trajectory is circular while experimental has the axis ratio of 1.3.

## Discussion

A continuous movement energy harvesting device with no friction parts has been proposed in the form of an orbiting circular blade fixed to an elastic beam. Due to the flow being always attached to the blade, high flow-to-mechanical power conversion coefficient, up to 3.5%, has been achieved.

Small-scale aeroelastic energy harvesters are convenient for powering wireless sensor nodes for structural health monitoring and environmental monitoring in smart cities, where replacing batteries is difficult and costly^21,22^. The orbiting blade conversion efficiency is lower than 6% of the galloping harvester, developed by the authors earlier^15^. However, the reported $C_p$ well exceeds that of a galloping square prism at 0.43%^23^ and vortex-induced vibration of circular cylinder systems at 0.07%^24^. A study summarising small scale wind turbines^25^ reports typical efficiency at the level of 20% at wind speeds of 10 m/s. Despite the efficiency of the orbiting blade being an order of magnitude less than that of small scale horizontal axis wind turbines and five times below that of Savonius rotors^26^, the absence of friction parts makes the system suitable for energy generation in harsh environments and remote locations, where the range of operating temperatures are high, abrasive dust/sand particles are present in the environment, and regular maintenance of friction parts is challenging.

An effect not observed in conventional turbines is the unstable shape of the trajectory, illustrated in Fig. 4. We attribute the instability of the orbital trajectory to the significant dependence of the wind energy conversion rate on the ratio of the blade velocity to the wind speed (i.e. on the angle of attack of the airfoil), and select only regimes with close to circular orbit for the measurement of the harvester performance. To illustrate how the orbit instability can be reduced, we note that in our experiments it is only observed in air, where the ratio of the beam-blade system kinetic energy to the energy harvested from the wind in one cycle is of the order of 10, while elliptic trajectories are not observed in water, where this ratio is less than a unit at 1/3. The energy provided by air/water at blade speeds when the orbit grows is comparable with that taken away when the orbit decays, making the ratios evaluated above similar to the damping factor for oscillations in perpendicular directions. Without performing a lengthy parametric study, a recommendation can be formulated for the harvesting systems operating in air: to avoid asymmetry of the orbiting trajectories, make the blade-beam system lighter.

Wind speed intermittency presents a key issue for real-world implementations of the orbiting blade, as with other types of wind energy harvesters, such as galloping and VIV. With the typical time of oscillation growth measured in tens of oscillation/orbiting cycles, the generation will stop when the wind drops below the generation threshold and may not recover during the next gust. This issue could potentially be resolved by the introduction of an asymmetry to the blade shape, resulting in a faster response to gusts.

Generation of electricity from the harvested wind energy is traditionally a difficult task for galloping and vortex-induced vibrations. The commonly used piezoelectric actuators are expensive and inefficient at frequencies at the level of few Hz, while high frequency wind energy harvesters are smaller and less efficient because of viscous losses. Here, continuous motion opens the way for electromagnetic generators, which still need adaptation to the configuration when the stator performs orbiting but does not rotate.

While the authors have used blades of simplistic shapes, single-part airfoils formed by two spherical surfaces, an obvious way to improve the aerodynamic efficiency is in designing profiles, suiting particular tip speed ratios and inclination of the blade normal to the incident flow velocity, or catering for the vicinity of the boundaries for devices installed in ducts. Ring-shaped blades, similar to the Chakram throwing weapon and Aerobi flying rings, can be used to decrease the induced drag. Geometries with multiple axisymmetric parts may be considered, but the analogy with slotted high-lift airfoils requires adjustment because the flow direction makes a full turn around the tip geometry.

## Conclusions

We have introduced a novel configuration of a continuous-movement aeroelastic energy harvester with no friction parts. The axisymmetric concave disk-shaped airfoil, fixed to an elastic beam, performs an orbiting motion perpendicular to the wind direction. At the appropriate orbiting speed, the flow is attached to the airfoil, resulting in the efficiency of the wind-to-mechanical energy conversion by the blade of up to 3.5%. In the configuration we used for experiments, aerodynamic and plastic losses in the beam reduced the overall efficiency to 1.8%. The experimentally obtained results have been successfully compared to numerical simulations.

In the present study, the airfoil geometry and beam parameters were selected based on intuitive considerations. As such, the current harvester configuration remains subject to optimisation in order to enhance efficiency, improve trajectory stability, and reduce sensitivity to wind direction and velocity fluctuations.

Similarly to conventional propellers, the action of energy production can be inverted to provide thrust by actuating the beam, turning the orbiting blade into a pump/propeller with no friction parts, suitable for applications in hostile environments.

### Captions to supporting videos

The Supporting video 1 is filmed in water at normal speed. The blade is covered with a lolly pop-like crystallised sugar with Methylene blue added as a dye. The flow velocity is 0.35 m/s, blade diameter 53 mm, orbit diameter 130 mm, orbiting frequency 1.65 Hz. The flow angle of attack on the plane of the blade rim is 25 degrees at the inner edge and 12 degrees at the outer edge.

The Supporting video 2, is a slowed down video filmed in the same conditions as the Supporting video 1 and aimed to illustrate that the flow remains attached to the blade surface.

## Supplementary Information

Supplementary Information 1.

Supplementary Information 2.

## Data Availability

The datasets used and/or analysed during the current study are available from the corresponding author on reasonable request. These datasets are time-resolved trajectories of the centre of orbiting blades tested in the wind tunnel.
